# Dentigerous Cyst associated with Impacted Maxillary Premolar: A Rare Site Occurrence and a Rare Coincidence

**DOI:** 10.5005/jp-journals-10005-1483

**Published:** 2017-02-01

**Authors:** Nitul Jain, Gaurav Gaur, Vivek Chaturvedy, Ajay Verma

**Affiliations:** 1 Associate Professor, Department of Oral Pathology, Eklavya Dental College & Hospital, Kotputli, Rajasthan, India; 2 Assistant Professor, Department of Oral and Maxillofacial Surgery, Eklavya Dental College & Hospital, Kotputli, Rajasthan, India; 3 Professor and Head, Department of Periodontology, Eklavya Dental College & Hospital, Kotputli, Rajasthan, India; 4 Professor and Head, Department of Oral and Maxillofacial Surgery, Eklavya Dental College & Hospital, Kotputli, Rajasthan, India

**Keywords:** Dentigerous cyst, Impacted premolars, Missing teeth, Nonsyndromic patient.

## Abstract

A dentigerous cyst is a developmental odontogenic cyst occurring most commonly in the third molar region of mandible and maxilla and maxillary canine region followed by mandibular premolar areas. However, dentigerous cyst occurring in association with impacted maxillary premolars is a very rare presentation accounting for less than 0.5% of total dentigerous cyst cases. In the following case report, a dentigerous cyst was reported in maxillary premolar region in a nonsyndromic patient having one missing premolar and another malformed one associated with the dentigerous cyst.

**How to cite this article:** Jain N, Gaur G, Chaturvedy V, Verma A. Dentigerous Cyst associated with Impacted Maxillary Premolar: A Rare Site Occurrence and a Rare Coincidence. Int J Clin Pediatr Dent 2018;11(1):50-52.

## INTRODUCTION

A dentigerous cyst is a developmental odontogenic cyst, which accounts for being the second only to radicular cyst in the chances of occurrence in oromaxillofacial region. A dentigerous cyst encloses the crown of an unerupted tooth or impacted tooth by expansion of the follicle attached to its neck^1^ caused by fluid accumulation between the reduced enamel epithelium and the enamel surface resulting into a cyst. The most common site for dentigerous cyst is the third molar region of mandible and maxilla and maxillary canine region followed by mandibular premolar areas. Lesion usually occurs in 2nd and 3rd decades with a slight male predilection.^2^

However, dentigerous cyst occurring in association with impacted maxillary premolars is a very rare presentation accounting for less than 0.5% of total dentigerous cyst cases reported in literature.^2^ The frequency estimated is about 1.44% of every 100 unerupted teeth.^3^ Along with this, a congenitally missing premolar in the same region adds up to more rarity in this case. Another thing that makes this case more interesting is that it was not possible to find out that the origin of dentigerous cyst was from 1st or 2nd premolar, as the impacted tooth had roots which were fused together, making it difficult to identify.

## CASE REPORT

A 12-year-old boy reported to the Department of Oral and Maxillofacial Surgery, Eklavya Dental College & Hospital, Kotputli, Rajasthan, India, with a chief complaint of asymptomatic painless swelling in the left maxillary anterior region since last 5 months. Extraorally hard swelling was palpable around left nasolabial fold, but no significant facial asymmetry was observed.

Clinical examination revealed a bony hard swelling in left maxillary vestibule area measuring about 3 × 4 cm, extending from maxillary left lateral incisor to 1st molar region. On palpation, the swelling was firm, painless, and consistent.

The overlying mucosa did not show any inflammatory signs. All permanent teeth were present except for maxillary left 1st and 2nd premolars. Both deciduous maxillary molars were present in 2nd quadrant with grade II mobility. No other carious lesion or periodontal condition was present.

**Fig. 1:**
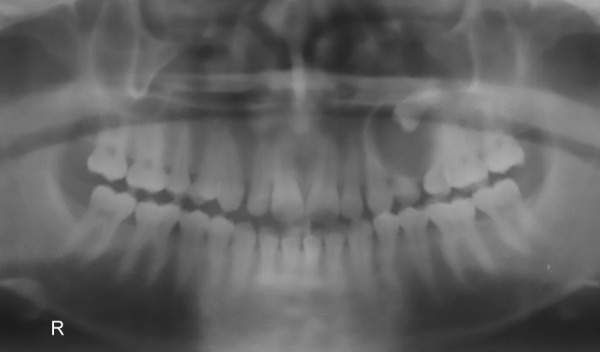
Preoperative orthopantomograph

Orthopantomograph (OPG) revealed a well-defined radiolucent lesion extending from distal aspect of canine to mesial aspect of first permanent molar with margins of lesion attached to the neck of one of the premolars (Fig. 1). Radiolucency included 1st and 2nd deciduous molars with complete root resorption and partial root resorption of canine. However, the canine was firm and vital on further examination. Another interesting finding was that the other premolar was absent in the maxillary left quadrant. It was difficult to comment whether it was the 1st or 2nd premolar missing or associated with the radiolucency.

**Fig. 2:**
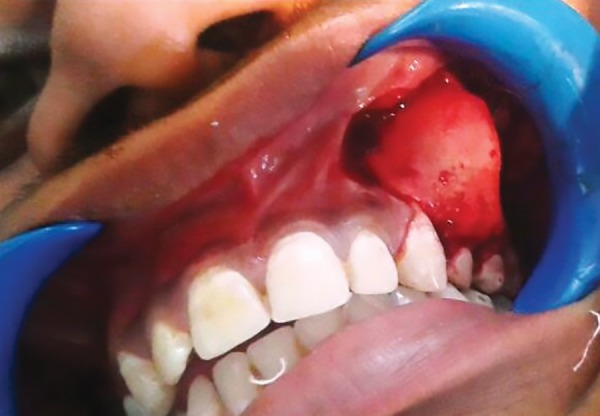
Expanded buccal cortex

**Fig. 3:**
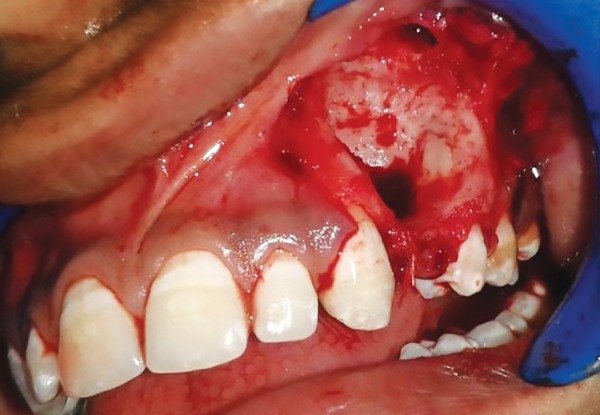
Enucleated cyst

**Fig. 4:**
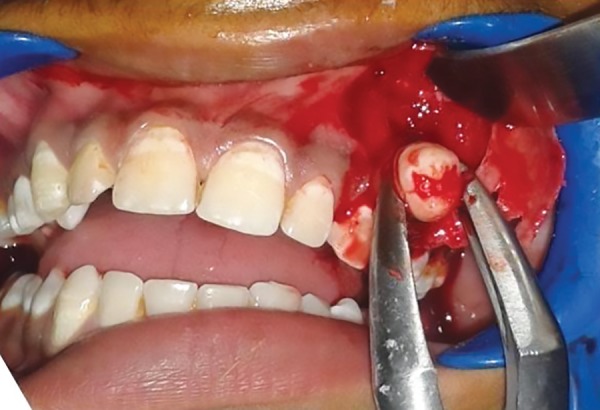
Impacted premolar

**Fig. 5:**
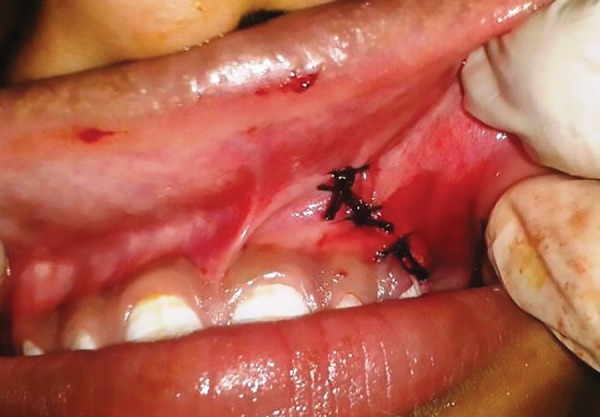
Closure

**Fig. 6:**
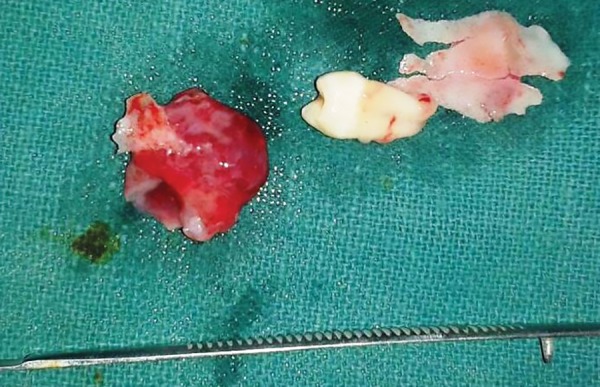
Specimen with tooth

To rule out the possible association of any syndrome complex, a thorough and complete systemic examination was performed by medical professionals; however, the patient was otherwise completely fine with no other significant systemic findings reported. Also, no other near or distant relative of patient had similar problems.

On aspiration of the lesion, a straw-colored fluid was aspirated, which provided us with a presumptive diagnosis of a cyst and ruled out the possibility of any odontogenic or nonodontogenic tumor.

With informed consent and under local anesthesia, both the deciduous molars were first extracted, and following this, a crevicular incision was given with an anterior release, extending from lateral incisor to 1st molar. Full thickness mucoperiosteal flap was elevated to expose the lesion (Figs 2 to 6). A paper-thin bone was present after the exposure which was later removed. The cystic lining was completely removed along with the impacted premo-lar, as the cystic lining was found attached to the neck of the tooth. Incision was closed using 3-0 black braded silk sutures and the specimen was sent for histopathological investigation. Histopathological analysis revealed dentig-erous cyst on microscopic examination. On microscopic examination, a cystic lining with three to four layers of nonkeratinized stratified squamous epithelium resembling reduced enamel epithelium with connective tissue capsule showing fibrous stroma with collagen fibers, blood vessels and few inflammatory cells were observed affirming the diagnosis of dentigerous cyst.

To restore the missing and extracted premolars, the patient was convinced for implant-supported crowns after bony healing following after 6 months.

## DISCUSSION

A dentigerous cyst is a cyst which usually encloses the crown of an unerupted tooth, expands the follicle, and is attached to the cementoenamel junction of the tooth. Most commonly, it involves the mandibular and maxillary third molar, maxillary permanent canines, followed by mandibular premolars and rarely maxillary premolars. The incidence rate of dentigerous cyst involving maxillary premolar is 2.7% as compared with 45.7% involving mandibular 3rd molar. Daley et al^3^ reported an incidence of 0.1 to 0.6% and Shear found it to be 1.5%. The present case report reveals the rare site of dentigerous cyst.

According to Toller et al,^4^ the origin of dentigerous cyst could be due to the breakdown of the proliferating cells of the follicle after impede eruption. There is an increase in osmotic pressure as a result of breakdown of products, finally leading to the development of cyst.^5^

A dentigerous cyst can be inflammatory or noninflammatory. The inflammatory type of dentigerous cyst occurs because of inflammation in a nonvital deciduous tooth. The noninflammatory type develops due to the pressure exerted by the erupting tooth on an impacted follicle.^6^ Radiographically, dentigerous cyst presents as well-defined unilocular radiolucency, often with a sclerotic border and this radiolucency surrounds the crown of an impacted tooth.^7,8^ Three radiographic variants of dentigerous cyst, namely central, lateral, and circumferential, have been described. In our case, the cyst was of central variety and presented as a unilocular radiolucency with a well-defined sclerotic border engulfing the crown of an impacted tooth.

Whenever the tooth involved in the dentigerous cyst is in a favorable position, it is preferable to perform marsupialization to facilitate the eruption of the tooth, but in our case, the impacted premolar was in an unusual position and root formation was also not complete, so enucleation was done along with extraction of the tooth.^9-12^

## CONCLUSION

Whenever young patients report with painless, slow, expansile lesions, especially with missing permanent teeth, benign odontogenic tumor or odontogenic cysts may be the reason behind their nonerupting conditions. That sometimes may be associated with a syndrome complex. Therefore, a complete clinical and radiological examination should be done in order to overcome certain confusing situations and render appropriate treatment.
